# A novel RFLP-PCR method for the rapid diagnosis of *Echinococcus multilocularis* and different *Echinococcus granulosus sensu lato* species from tissue samples

**DOI:** 10.1051/parasite/2026017

**Published:** 2026-04-01

**Authors:** Gérald Umhang, Jean-Marc Boucher, Lisa Laboutière, Grégory Karadjian

**Affiliations:** 1 INTERFAS Unit, ANSES, National Reference Laboratory for Echinococcus spp., Rabies and Wildlife Laboratory 54220 Malzéville France; 2 UMR BIPAR, ANSES, Laboratoire de Santé Animale, INRAE, École Nationale Vétérinaire d’Alfort 94700 Maisons-Alfort France

**Keywords:** *Echinococcus*, Molecular diagnostic, RFLP-PCR, Larval stage

## Abstract

Both alveolar echinococcosis and cystic echinococcosis are zoonotic diseases affecting thousands of humans in Europe annually. Five *Echinococcus* species are known to be endemic in Europe, but with varying relative importance in humans and animals. In order to identify these *Echinococcus* species from tissue samples, an RFLP-PCR method was developed and validated in accordance with the French quality standards for molecular diagnostics (NF U47-600-2). Amplification by PCR of about 846 bp from the 12S ribosomal RNA gene was followed by a single digestion step with three restriction enzymes (AccI, DdeI and HinfI). The RFLP-PCR method distinguishes *Echinococcus multilocularis*, *E. granulosus sensu stricto*, *E. equinus*, *E. ortleppi* and *E. canadensis* in six specific profiles composed of two or three bands. Each profile is specific for the species, except an additional specific profile from genotype G8 of *E. canadensis.* Species from other genera of the Taeniidae family correspond to two other types of profiles. Additionally, *in silico* analyses predicted three specific additional profiles for the other four *Echinococcus* species. The limit of detection of the method including DNA extraction is estimated to be a minimum of 1,000 copies. Total specificity and sensitivity were obtained by testing a panel of 73 and 79 DNA samples previously identified by sequencing. This RFLP-PCR technique is cost-effective, simple and rapid to implement, making it suitable for use in large epidemiological studies, as well as for single diagnostic purposes targeting tissue samples, such as cysts or worms, without requiring sequencing.

## Abstract (fr)

L’échinococcose alvéolaire et l’échinococcose kystique sont des zoonoses qui affectent chaque année des milliers de personnes en Europe. Cinq espèces d’*Echinococcus* sont endémiques en Europe, mais leur importance relative chez l’homme et l’animal varie. Afin d’identifier ces espèces d’*Echinococcus* à partir d’échantillons tissulaires, une méthode RFLP-PCR a été développée et validée conformément aux normes françaises de qualité pour le diagnostic moléculaire (NF U47-600-2). L’amplification par PCR d’un fragment d’environ 846 pb du gène de l’ARN ribosomique 12S est suivie d’une digestion unique par trois enzymes de restriction (AccI, DdeI et HinfI). La méthode RFLP-PCR permet de distinguer *Echinococcus multilocularis*, *E. granulosus sensu stricto*, *E. equinus*, *E. ortleppi* et *E. canadensis* grâce à six profils spécifiques composés de deux ou trois bandes. Chaque profil est spécifique de l’espèce, à l’exception d’un profil spécifique supplémentaire associé au génotype G8 d’*E. canadensis*. Les espèces appartenant à d’autres genres de la famille des Taeniidae correspondent à deux autres types de profils. De plus, des analyses bioinformatiques ont prédit trois profils spécifiques supplémentaires pour les quatre autres espèces d’*Echinococcus*. La limite de détection de la méthode, extraction d’ADN incluse, est estimée à au moins 1 000 copies. La spécificité et la sensibilité totales ont été obtenues en testant un panel de 73 et 79 échantillons d’ADN préalablement identifiés par séquençage. Le faible coût, la simplicité et la rapidité de mise en œuvre de cette technique RFLP-PCR la rendent adaptée aux études épidémiologiques de grande envergure, ainsi qu’au diagnostic ciblé d’échantillons tissulaires, tels que des kystes ou des vers, sans nécessiter de séquençage.

## Introduction

The genus *Echinococcus*, which belongs to the family Taeniidae, is currently composed of nine different species [20, 41]. The most common species are *Echinococcus multilocularis* and those of *Echinococcus granulosus sensu lato* (*s.l*.). The latter is currently recognised as a complex of five species: *Echinococcus granulosus sensu stricto* (*s.s*.), *E. equinus*, *E. ortleppi*, *E. canadensis* and *E. felidis*. The taxonomy of *E. canadensis* remains unresolved, with four genotypes (G6, G7, G8 and G10) grouped together, which may be divided into two or three distinct species [18, 26, 29]. With the exception of *E. felidis*, which is exclusively distributed in Africa, all other species of *E. granulosus s.l.* are present in Europe as *E. multilocularis* and have been shown to be zoonotic, but with varying relative importance.

*Echinococcus multilocularis* and *E. granulosus s.l.* are the etiological agent of alveolar echinococcosis (AE) and cystic echinococcosis (CE), respectively. Both are chronic diseases with initial asymptomatic periods that can last up to 15 years. Compared to CE, which is a disabling disease with a low mortality rate, AE is more severe and has a high mortality rate without treatment [4]. The average annual incidence of AE in humans in the 27 Member States of the European Union was 151 cases per year between 2018 and 2022 [7]. From 1997 to 2021, 54,244 cases of human CE were identified in the 27 Member States [5]. The distinction between AE and CE in humans is typically made on the basis of imaging and serological techniques rather than molecular diagnostics, which is not always possible, and which is not consistently reported in official statistics. Furthermore, the identification of the *E. granulosus s.l.* species involved in CE cases, which necessarily requires molecular biology, is even less systematic. In this context, a systematic review of 599 molecularly confirmed human CE cases from 29 European countries between 2000 and 2021 showed that *E. granulosus s.s.* was the most common species (76.8%), followed by the *E. canadensis* cluster (21.7%) and *E. ortleppi* (1.2%) [6]. The recent identification of the first human case due to *E. equinus* has raised questions about its potential for transmission to humans [22].

In Europe, *E. multilocularis* is maintained by a sylvatic lifecycle based on the predation of voles (*Microtus arvalis* and *Arvicola* spp.) by red foxes. A significant number of mammals, acting as either intermediate or dead-end hosts, have been identified as having the potential to develop AE. These include aquatic rodents, pigs, wild boars, horses, and a range of other exotic species commonly found in zoos or wildlife parks, such as lemurs and monkeys [25]. The European species of *E. granulosus s.l.* mainly have domestic life cycles involving dogs and livestock. Although other canid species, particularly wolves, can act as definitive hosts, there is typically a predominance of one type of intermediate host species for each *E. granulosus* species. *Echinococcus granulosus s.s.* is of primary concern for sheep, but it may also infect a wide range of other intermediate hosts in livestock, including cattle and pigs. In addition, it can infect wildlife species, including wild boar and cervids. Additionally, *E. equinus* and *E. ortleppi* are more specific and are primarily associated with equids (horses and donkeys) and cattle, respectively. Genotype G7 of *E. canadensis* is primarily associated with pigs and wild boar in Europe, while G6 concerns camels in North Africa, the Near and Middle East, and Central Asia. In contrast, genotypes G8 and G10, which are present only in northern Eurasia, have been observed to infect wild cervid species (moose, elk/wapiti and roe deer) as intermediate hosts.

The progress made in the field of molecular biology over the past four decades has been pivotal in the diagnosis of *Echinococcus* species from tissue samples. The morphological identification of worms has typically enabled differentiation between *E. multilocularis* and *E. granulosus s.l.* However, distinguishing between the various species within the *E. granulosus* complex has remained challenging, particularly given that some variations may be attributed to the definitive host species and the quality of the samples, which can often be a limitation when dealing with wildlife samples. Furthermore, the diagnostic process at the species level proved particularly challenging with regard to the larval stage. It was not possible to distinguish between species in intermediate or accidental hosts, particularly within the *E. granulosus* complex, either in animal or human samples. Molecular methods are currently regarded as the most pertinent approaches, with polymerase chain reaction (PCR) techniques targeting a single species or multiplex PCR representing the most effective techniques. Nevertheless, sequencing is typically conducted on the amplicons obtained from the initial PCR to confirm the species or to identify the species targeted by the PCR. This is exemplified by the commonly used Cest Multiplex from Trachsel *et al.* [30] in order to identify the *E. granulosus* species. One of the most common techniques is the use of PCR to amplify a fragment of the mitochondrial genome (*e.g.*, *cox1*, *nad1* and *nad5*) using primers efficient for a wide range of parasite species. The required sequencing to identify the *Echinococcus* species in question increases the time needed for analysis, as well as the associated costs. The cost, in turn, presents a barrier to use of the technique in low-income countries, yet it is often required for more accurate characterization of the lifecycles involved. The restriction fragment length polymorphism-PCR (RFLP-PCR) method offers several advantages, including rapidity, simplicity and cost-effectiveness due to the generation of a wide range of specific band patterns following electrophoresis.

Since the 1990 s, a number of RFLP-PCR assays have been developed for use with *Echinococcus* species. The primary differentiating factor between these tests is the target species, which corresponds to disparate epidemiologic scenarios. For example, the ability to distinguish between *E. multilocularis*, *E. granulosus s.s.* and *E. shiquicus* was particularly important for understanding the epidemiology of echinococcosis on the Tibetan plateau of China [43]. Similarly, the approach developed by Hüttner *et al.* [11] enabled the identification of *E. felidis* among other *E. granulosus* species from taeniid eggs isolated in African wild carnivores and cysts of livestock species. Globally, RFLP-PCR assays have focused primarily on distinguishing between *E. granulosus* species, with *E. multilocularis* often omitted, even in the context of endemic European populations [9, 27, 28]. This article presents a novel RFLP-PCR diagnostic method targeting all five endemic European *Echinococcus* species. The methodology has been validated in accordance with the French quality standards for molecular diagnostics (NF 47-600-2).

## Materials and methods

### Parasitic material

A genomic DNA reference sample from *E. multilocularis* was used for the purpose of evaluating the methodology. This reference DNA was obtained from the metacestode stage of a parasite strain, which was maintained experimentally on Swiss EOPS mice (APAFIS #34612-2022011114104529). The DNA from this parasitic tissue was extracted manually using a standard tissue kit (QIAGEN, Hilden, Germany). The number of mitochondrial genome copies was determined using a specific real-time PCR for *E. multilocularis* [16], as described by Umhang *et al.* [32]. To evaluate method sensitivity and specificity, DNA from field samples of different *E. multilocularis* and *E. granulosus s.l*. species, as well as from other genera within the Taeniidae family (*Taenia*, *Hydatigera* and *Versteria*), which were already available at the laboratory, were used. Prior to the present study, all samples underwent species identification through short *cox1* sequencing, as described by Bowles *et al.* [3].

### RFLP-PCR method

In the context of method evaluation, DNA extraction was performed using a commercially available kit (Maxwell RSC Blood DNA Kit) with the Maxwell48 automatic extractor (Promega, Madison, WI, USA) based on purification with magnetic particles. A piece of tissue weighing approximately 10–20 mg was initially digested with proteinase K. Following centrifugation, the supernatant was transferred to the first well of the cartridge, which was then placed in the instrument for the subsequent washing steps, concluding with elution in 100 μL.

The two PCR primers Taen F (5′–GTTTGCCACCTCGATGTTGACT–3′) and ITMTn R (5′–CTCAATAATAATCGAGGGTGACGG–3′) used in this method were initially published by Geysen *et al.* 2007 [8] and reported to be specific to the *Taenia* genus in the context of identification of *Taenia saginata* in muscle lesions by RFLP-PCR. Nevertheless, while amplification of about 846 bp from the 12S ribosomal RNA is obtained for species of the *Taenia* genus, it is also possible for other species of the Taeniidae family, including the *Echinococcus* genus. The reaction mixture consisting of 5 μL of 10× PCR buffer (Invitrogen, Carlsbad, CA, USA), 1.5 μL of 50 mM MgCl_2_ solution, 1 μL of 10 mM dNTP mix, 2.5 μL of each primer (Taen F and ITMTn R, 10 μM each), 0.2 μL of Platinum Taq DNA polymerase, 3 μL of DNA template and enough nuclease-free water was prepared to reach 50 μL of final volume for each reaction. The protocol for PCR amplification was initiated with a denaturation step at 94 °C (2 min), followed by repetition of 35 cycles composed of denaturation at 94 °C (45 s), annealing at 57 °C (45 s) and extension at 72 °C (60 s), ending with final extension at 72 °C (7 min). The PCR products were separated on a 1.5% agarose gel stained with SYBR Safe DNA Gel Stain (Invitrogen). A negative control (3 μL of nuclease-free water) was run in agarose gel with the RFLP products and positive controls composed of 3 μL of suspected *Echinococcus* species DNA. After confirmation of the presence a PCR product, 6 μL of the PCR product of each sample were incubated for 3 h at 37 °C with 9 μL of a mixture containing AccI, DdeI and HinfI restriction enzymes (corresponding to 2.7 units for each of the three) and 1.3 μL of 10× rCutSmart™ Buffer (New England Biolabs, Ipswich, MA, USA) in 7.5 μL of nuclease-free water. The restriction fragments were separated by electrophoresis in 3% MetaPhor agarose gel (Lonza) for 45 min at 110 V for medium size (15 cm). A DNA size marker of 100 bp was included for size identification of the bands with Uvitec 1D software (Uvitec Ltd., Cambridge, UK). In the absence of amplification, the DNA extracts were diluted (1/10) and reamplified.

### Validation of the RFLP-PCR method

#### Limit of detection

The limit of detection (LOD) of the PCR is the smallest number of copies of target nucleic acid per unit volume that can be detected in 95% of cases. To determine this limit, three independent ranges of six different dilutions were used. For each range point, 8 replicates were performed, yielding 24 results per assay point tested. The approximate value of the LOD corresponds to the dilution giving at least 23 positive results out of 24. The LOD of the full method (DNA extraction followed by RFLP-PCR) corresponds to the minimum quantity of biological target that must initially be present in the sample in order to obtain an interpretable profile. The test sample matrix corresponds to 20 mg liver tissue sample from a naïve Swiss EOPS mouse spiked with a different number of copies of the *E. multilocularis* reference DNA. Each extracted DNA was tested 8 times in two independent sessions for each of the dilutions tested. The LOD was estimated as the last dilution level where all 8 replicates are positive.

#### Specificity and sensitivity evaluation

Diagnostic specificity was estimated from 73 tissue samples corresponding to suspicion of *E. multilocularis* or *E. granulosus sensu lato* which was diagnosed as negative (*n* = 32: no amplification by short *cox1* PCR or non-parasitic result after sequencing) or corresponding to infection by Taeniidae species other than from the *Echinococcus* genus (*n* = 41: *Taenia hydatigena*, *T. krabbei*, *T. polyacantha*, *Hydatigera kamiyai* and *Versteria mustelae*) due to short *cox1* sequence analysis (Table 1). Diagnostic sensitivity was estimated from 79 positive tissue samples from the laboratory collection for which the presence of *E. multilocularis* or an *E. granulosus s.l.* species (*E. granulosus sensu stricto*, *E. equinus*, *E. ortleppi* and *E. canadensis*) had previously been confirmed. Regarding *E. multilocularis*, samples representing the four different clades (European, Asian, North American and Mongolian) were tested as the different genotypes of *E. granulosus s.l*. (Table 2). Regarding *Echinococcus* species not available at the laboratory (*E. shiquicus*, *E. vogeli* and *E. oligarthra*), an *in silico* analysis was carried out using Geneious prime software to obtain the expected profile bands.

**Table 1:** List of samples according to parasite and host species used to evaluate the specificity of the PCR-RFLP assay. The asterisk indicates worms as the matrix, while all the other samples concern the larval stage.

Parasite species	Host species	No. of samples	Reference
/	mouflon	1	/
	ferret	1	/
	cattle	30	[37]
*H. kamiyai*	muskrat	4	[38]
*T. hydatigena*	cattle	2	[37]
	goat	1	[37]
	sheep	5	[37]
*T. krabbei*	roe deer	1	/
	grey wolf*	1	[34]
*T. polyacantha*	muskrat	4	[38]
	nutria	2	[38]
*V. mustelae*	bank vole	12	[36]
	muskrat	5	[38]
	nutria	4	[38]

**Table 2:** List of samples corresponding to the five *Echinococcus* species targeted by the PCR-RFLP assay in the context of sensitivity evaluation. The asterisk indicates worms as the matrix, while all the other samples concern the larval stage.

Parasite species	Clade or genotype	Host species	Origin	No of samples	Reference
*E. multilocularis*	European	vole bank	France	5	[36]
	European	terrestrial vole	France	2	[36]
	European	red fox*	France	21	/
	North American	arctic fox*	Norway (Svalbard)	5	/
	Mongolian	Olkhon mountain vole	Russia	2	[31]
	Asian	red fox*	Poland	5	[13, 35]
*E. granulosus sensu stricto*	G3	sheep	France	4	[37]
	G1	sheep	Moldova	4	[33]
	G1	sheep	Morocco	4	[2]
	G3	chamois	France	1	[39]
*E. equinus*	/	horse	United Kingdom	3	/
*E. ortleppi*	/	cattle	France	5	[10, 37]
*E. canadensis*	G6	camel	Mongolia	3	[1]
	G6	camel	Mauritania	6	[2]
	G7	pig	France	7	[40]
	G8	moose	Russia	1	[17]
	G10	reindeer	Russia	1	[42]

**Table 3:** RFLP banding patterns for all the *Echinococcus* species (genotypes concerned indicated in parentheses) and other taeniid species tested obtained after digestion by DdeI, HinfI and AccI restriction enzymes of the 12S ribosomal RNA amplicon. The various bands are distinguished from one another by a slash, whereas fragments that are not identical but of comparable size, which subsequently form a single band following electrophoresis, are grouped together in brackets. For species marked with an asterisk, the *in silico* band profile could not be validated by a biological test.

Parasite species	Banding patterns in bp
*E. multilocularis*	447/137/(125–126)
*E. granulosus s.s.* (G1, G3)	324/249/125
*E. equinus*	571/(123–126)
*E. ortleppi*	330/251
*E. canadensis* (G6, G7)	312/ (250–251)
*E. canadensis* (G10)	309/ (247–248)
*E. canadensis (G8)*	329/287/247
*E. felidis	247/191/(122–123–127)
*E. oligarthra	323/233/(122–124)
*E. vogeli	327/247/(122–123)
*E. shiquicus	541/(122–124)
Other taeniid species tested	515–560/230–300

#### Method robustness

The robustness of the method was evaluated by testing variations of different conditions in the amplification of *E. multilocularis* reference DNA. A variation in annealing temperature from 61 to 71 °C by increments of 2 °C was tested with quantities of DNA ranging from 0.1 to 10 ng. Concentrations of primers from 0.25, 0.5, 1 and 1.5 mM and from 0.5, 1, 1.5 and 2 mM of magnesium were tested with 0.1 ng of *E. multilocularis* DNA. The potential impact of variation in the enzymatic digestion temperature was evaluated from 33 to 41 °C for 0.1 to 10 ng of *E. multilocularis* reference DNA.

## Results

### Description of the *Echinococcus* specific profiles

**Figure 1:**
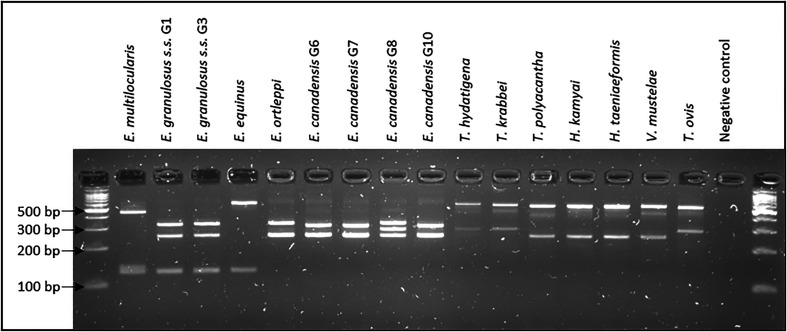
RFLP profiles from *E. multilocularis*, *E. granulosus* species and other taeniid species obtained after digestion by the AccI, DdeI and HinfI restriction enzymes of the PCR product of about 846 bp from the 12S ribosomal RNA gene.

The RFLP-PCR method distinguishes *E. multilocularis*, *E. granulosus s.s*., *E. equinus*, *E. ortleppi* and *E. canadensis* in six specific profiles composed of two or three bands (Fig. 1 and Table 1). Each profile is specific for the species, except the profile from genotype G8 of *E. canadensis* which was different from the one obtained for the other genotypes (G6, G7 and G10) of this species. Based on *in silico* analyses, the profile of genotype G10 is slightly different (3 bp) from genotypes G6 and G7 but appears identical after electrophoresis. Species from other genera of the Taeniidae family correspond to two other types of profiles composed of two bands. Additionally, *in silico* analyses predicted three specific additional profiles for the four other *Echinococcus* species. The same profile is expected for *E. vogeli* and *E. oligarthra*, while two others are obtained for *E. felidis* and *E. shiquicus*. Globally, 9 different profiles were obtained for the 9 *Echinococcus* species (including G1 and G3 genotypes for *E. granulosus s.s.* and the four genotypes of *E. canadensis*) and two others for the other Taeniidae species.

### Limit of detection

Regarding the LOD of the PCR, positive results were obtained for all 8 replicates in each of the 3 independent assays for number of copies corresponding to 3,000, 1,200 and 600. Regarding the lower number of copies, detection among the 24 replicates was 23 and 19 for 300 and 150 copies, respectively. The LOD of the PCR is estimated at 300 copies. Considering the complete method, an interpretable RFLP profile was obtained in all 8 replicates for the different number of copies tested: 500,000, 100,000, 10,000 and 1,000. The LOD is estimated to be at a minimum of 1,000 copies.

### Specificity and sensitivity

No amplification was obtained regarding the 32 samples corresponding to non-parasitic infection. Two types of profiles were observed from Taeniidae species other than *Echinococcus* sp.: one grouping *T. hydatigena*, *T. krabbei* and *T. ovis* and the other one grouping *T. polyacantha*, *H. kamyai*, *H. taeniaeformis* and *V. mustelae*. These two profiles differed largely from those expected from *E. multilocularis* and *E. granulosus s.l*. Regarding the samples tested, the RFLP-PCR test is considered to be specific for the five *Echinococcus* species tested with the expected specific profiles. There was no difference in profiles whether for the different clades of *E. multilocularis* or between genotypes of the same species of *E. granulosus s.l.*, except for genotype G8 of *E. canadensis*.

### Robustness of the PCR-RFLP method

No visible variation in the amplification was observed based on changes in primer concentrations, annealing and enzymatic digestion temperatures. Regarding MgCl_2_ concentrations, no amplification was observed at a concentration of 0.5 mM and a slightly lower amplification at 1 mM.

## Discussion

The RFLP-PCR method described in this study allows for the straightforward and rapid identification of the five most common *Echinococcus* species from tissue samples in a single assay, avoiding the need for sequencing. This is achieved by obtaining specific RFLP profiles following the amplification of a cestode-specific region of the 12S mitochondrial gene, which is then subjected to an enzymatic digestion step. The remaining four *Echinococcus* species (*E. vogeli*, *E. oligarthra*, *E. shiquicus* and *E. felidis*), which have a more restricted geographical distribution, can also be identified based on *in silico* analyses. Two additional profiles were generated for the genera *Taenia*, *Hydatigera* and *Versteria*.

The cost-effective, simple and rapid implementation of this RFLP-PCR technique makes it suitable for use in large epidemiological studies, as well as for single diagnostic purposes targeting tissue samples, such as cysts or worms. Evaluation of the method yielded highly promising results. A high concentration of parasite DNA is typically obtained from cysts and worms, with an estimated 7,000 copies of the mitochondrial genome present in a single taeniid egg [30]. The LOD of the complete method, including DNA extraction, is therefore of particular significance in the context of these tissue matrices, with the ability to detect 1,000 and 300 copies when considering solely the RFLP-PCR step. The *Echinococcus* profiles were consistently generated from the *Echinococcus* samples tested, thereby confirming the method’s high sensitivity and specificity. This finding was corroborated by *in silico* results from other *Echinococcus* species and the two additional profiles obtained from the other five Taeniidae species tested. The Taeniidae species selected for testing are a representative selection of the most commonly encountered species in the context of animal tissue diagnostics. Of note, *T. hydatigena* is commonly found in livestock species and, to a lesser extent, in wild mammal herbivores that may also be infected with *E. granulosus s.l*. Also, *T. krabbei* was selected because its lifecycle is generally completed between wolves and deer, which may also be infected with *E. granulosus s.s.* and, more specifically, the G8 and G10 genotypes of *E. canadensis*. Rodents are often infected by the larval stage of *H. kamyai*, *V. mustelae* and *T. polyacantha*. It should be noted, however, that infection by *E. multilocularis* is also a possibility. It is very important epidemiologically to be able to distinguish this latter zoonotic species from those previously mentioned.

Recently, real-time PCR multiplex assays have been developed for the diagnosis of metacestode infections, with particular focus on humans. These assays facilitate the specific detection of *E. multilocularis*, *E. granulosus s.l*., as well as *Taenia* sp. and *Toxocara* sp. [15, 24]. However, the use of multiplex real-time PCR requires the use of numerous primer pairs and probes, which increases the cost and complexity of the process. Moreover, the need for dedicated, expensive equipment represents a significant additional challenge in some parts of the world. In comparison, the RFLP-PCR assay has the advantage of simplicity and low cost, requiring only one PCR amplification and one digestion step to directly identify the *Echinococcus* species. The cost of the method from PCR amplification to electrophoresis, including digestion, was estimated in our conditions to be €1.89 for one sample. Other methods of identifying *Echinococcus* species based on multiplex PCR or including RFLP have previously been published in the scientific literature. However, these methods require successive PCR or sequencing steps for at least some targeted species [28, 30]. The RFLP-PCR method described here eliminates the need for sequencing in order to obtain species-level identification. Nevertheless, it may be feasible to identify species other than *Echinococcus* in the Taeniidae family by analysing the sequence of the 12S gene fragment. With regard to *Echinococcus* species, sequencing may be needed for identification at the genotype involved in *E. granulosus s.s.* or *E. canadensis*. Nevertheless, in these specific cases, it is recommended that the previously identified mitochondrial genes (*nad5* and potentially *nad2*) be sequenced in order to guarantee consistent identification [14, 19].

The method was primarily developed for use in the European context, with the specific aim of targeting the endemic *Echinococcus* species. Nevertheless, this method can be employed in other epidemiologic contexts, particularly in light of the findings from the *in silico* analyses for the remaining four *Echinococcus* species. A potential limitation of this RFLP-PCR approach is the need for sequencing in instances of low-quality electrophoresis, particularly for the differentiation between closely related profiles. This may be the case for *E. canadensis* and *E. ortleppi*, but also to distinguish between *E. equinus* and *E. shiquicus*, although the latter would be restricted to samples originating from the Tibetan Plateau, which is the endemic area of *E. shiquicus*. The use of control DNA of the relevant *Echinococcus* species notably *E. ortleppi* and *E. canadensis* G6/G7 would prevent potential confusion. Moreover, the method was not evaluated for copro-DNA, as this is not recommended, particularly in light of the prevalence of co-infections, which would result in complex and uninterpretable profiles. With regard to the detection of pathogens in this complex matrix, a number of real-time copro PCR assays have recently been developed, and these appear to be more relevant for copro-DNA [12, 16, 21, 23].

## Conclusion

This RFLP-PCR method, based on a single PCR reaction, followed by a single digestion step, enables the specific identification of the five principal *Echinococcus* species from animal or potentially human tissue samples. Furthermore, it allows for the identification of other taeniid species after an additional sequencing step. The method has been demonstrated to be highly specific, even when applied to the four rarer *Echinococcus* species. The method is rapid, simple, robust, sensitive and specific, and is therefore suitable for use in routine and large-scale studies of tissue samples, particularly in Europe, but potentially also on other continents with different epidemiologic situations.
